# Countrywide insecticide resistance monitoring and first report of the presence of the L1014S knock down resistance in Niger, West Africa

**DOI:** 10.1186/s12936-022-04410-4

**Published:** 2022-12-16

**Authors:** Hadiza Soumaila, Boubé Hamani, Ibrahim Issa Arzika, Amadou Soumana, Abdoulaye Daouda, Fatoumata Abdoulaye Daouda, Souleymane Mahaman Iro, Samira Gouro, Maman Sani Zaman-Allah, Izamné Mahamadou, Saadou Kadri, Noura Maman Salé, Wilfried Hounkanrin, Boubacar Mahamadou, Halima Naroua Zamaka, Rabiou Labbo, Ibrahim Maman Laminou, Hadiza Jackou, Sabiti Idrissa, Eric Coulibaly, Zilahatou Bahari-Tohon, Els Mathieu, Jenny Carlson, Ellen Dotson, Taiwo Samson Awolola, Cecilia Flatley, Joseph Chabi

**Affiliations:** 1 PMI VectorLink Project, Niamey, Niger; 2 National Malaria Control Programme, Niamey, Niger; 3 Centre de Recherche Médicale et Sanitaire, Niamey, Niger; 4 U.S. President’s Malaria Initiative, USAID, Niamey, Niger; 5 Entomology Branch, U.S. President’s Malaria Initiative, Atlanta, GA USA; 6 U.S. Centers for Disease Control and Prevention, Atlanta, GA USA; 7 PMI VectorLink Project, Washington, DC USA

**Keywords:** Insecticide resistance, Resistance mechanisms, New generation ITNs, Malaria, Niger

## Abstract

**Background:**

Mass distribution of insecticide-treated nets (ITNs) is the principal malaria vector control strategy adopted by Niger. To better inform on the most appropriate ITN to distribute, the National Malaria Control Programme (NMCP) of Niger and its partners, conducted insecticide resistance monitoring in selected sites across the country.

**Methods:**

The susceptibility of *Anopheles gambiae *sensu lato (*s.l*.) to chlorfenapyr and pyrethroid insecticides was investigated in a total of sixteen sites in 2019 and 2020, using 2–5-day-old adults reared from wild collected larvae per site. The susceptibility status, pyrethroid resistance intensity at 5 and 10 times the diagnostic concentrations, and piperonyl butoxide (PBO) synergism with diagnostic concentrations of deltamethrin, permethrin and alpha-cypermethrin were assessed using WHO bioassays. Two doses (100 and 200 µg/bottle) of chlorfenapyr were tested using the CDC bottle assay method. Species composition and allele frequencies for knock-down resistance (*kdr*-L1014F and L1014S) and acetylcholinesterase (*ace-1* G119S) mutations were further characterized using polymerase chain reaction (PCR).

**Results:**

High resistance intensity to all pyrethroids tested was observed in all sites except for alpha-cypermethrin in Gaya and Tessaoua and permethrin in Gaya in 2019 recording moderate resistance intensity. Similarly, Balleyara, Keita and Tillabery yielded moderate resistance intensity for alpha-cypermethrin and deltamethrin, and Niamey V low resistance intensity against deltamethrin and permethrin in 2020. Pre-exposure to PBO substantially increased susceptibility with average increases in mortality between 0 and 70% for tested pyrethroids. Susceptibility to chlorfenapyr (100 µg/bottle) was recorded in all sites except in Tessaoua and Magaria where susceptibility was recorded at the dose of 200 µg/bottle. *Anopheles coluzzii* was the predominant malaria vector species in most of the sites followed by *An. gambiae *sensu stricto (*s.s*.) and *Anopheles arabiensis*. The *kdr*-L1014S allele, investigated for the first time, was detected in the country. Both *kdr*-L1014F (frequencies [0.46–0.81]) and L1014S (frequencies [0.41–0.87]) were present in all sites while the *ace-1* G119S was between 0.08 and 0.20.

**Conclusion:**

The data collected will guide the NMCP in making evidence-based decisions to better adapt vector control strategies and insecticide resistance management in Niger, starting with mass distribution of new generation ITNs such as interceptor G2 and PBO ITNs.

**Supplementary Information:**

The online version contains supplementary material available at 10.1186/s12936-022-04410-4.

## Background

Malaria is one the leading health concerns in endemic countries and particularly in sub-Saharan Africa [1, 2]. Several negative outcomes, including disability, death, and slow economic growth, are attributed to malaria. However, measures to prevent and/or treat the disease are limited and costly [3]. For decades, mass distribution of insecticide-treated nets (ITNs) and indoor residual spraying (IRS) have represented the core malaria prevention strategies recommended by the World Health Organization (WHO) and have been implemented in many endemic countries [4]. In recent years, the development and spread of insecticide resistance in malaria vectors has become an increasingly important threat to vector control and disease prevention efforts [5–12].

In Niger, malaria is endemic and is the primary cause of illness, death and disability, disproportionately affecting children under 5 years of age. It accounts for 28% of all illness in the country and 50% of all recorded deaths [2]. The National Epidemiological Report of the National Malaria Control Programme (NMCP) indicates that there were 4,490,328 cases and 4170 malaria deaths in 2021, putting the country among those with the highest per capita rates of malaria fatalities globally [13]. In 2019, 29 countries accounted for 95% of malaria cases worldwide, among which five countries including Niger accounted for about 51% of all cases globally [2].

The estimated number of cases decreased by 7.9% between 2015 and 2019 (from 204 cases per 1000 population to 131 per 1000 of the population at risk) and the number of deaths decreased by 25.9% in the same period (from 0.919 deaths per 1000 population to 0.730 deaths per 1000 of the population at risk) [14]. ITN distribution represented the main vector control strategy that was implemented in the country, coupled with diagnosis and treatment of malaria cases. Niger conducted mass ITN distributions in 2005, 2009, 2014 and 2017 targeting different regions across the country and guided by ‘high burden high impact (HBHI)’ approaches during each campaign, in addition to the routine distribution for pregnant women and children under the age of 5 years. The country adopted the HBHI recommended by the WHO to target higher endemic areas within the country, by deploying massive vector control interventions and malaria care, and empowering politics for a coordinated response to the disease using relevant data collection and use for appropriate decision-making [15]. To protect children under the age of 5 during the peak transmission season, the NMCP has also been conducting annual seasonal malaria chemoprevention (SMC) since 2013 and has reached yearly more than 4 million children across 58 of the 61 eligible districts. Additional measures to combat malaria in Niger included intermittent preventive treatment during pregnancy (IPTp), and case management.

In 2020, the Niger NMCP reviewed the country’s malaria risk map to allow the selection of priority interventions according to the endemicity level and to align interventions with the HBHI approaches. Therefore, the country was divided into four new endemicity strata including a very low transmission strata, also referred to as sporadic malaria cases and characterized by an incidence of less than 100 cases per 1000 population, low transmission representing areas of > 100 and < 250 cases per 1000 population, moderate transmission with > 250 and < 450 cases per 1000 population, and a high transmission strata recording more than 450 cases per 1000 population where the highest incidences were recorded, considering both malaria cases among children under five years of age and transmission data collected over the last 5 years. The vast majority of the population (94%) resides in the two southernmost (moderate and high transmission) zones where malaria is most prevalent.

Previous entomological activities conducted in the country have already highlighted malaria control challenges, including insecticide resistance in the main malaria vectors in the country [16–18]. Since 2018, the U.S. President’s Malaria Initiative (PMI) has supported Niger’s NMCP and its partners to conduct insecticide susceptibility testing in several sites across the country to help update the country’s malaria vector control strategy and selection of control tools.

## Methods

### Study sites

Niger is a West African country covering a land area of almost 1,270,000 km^2^, making it the largest country in West Africa with a population of about 22 million living mostly in clusters in the far south and west of the country. Over 80% of its land area lies in the Sahara Desert. The climate is mainly very dry and very hot with a peak temperature of about 45 °C between January and February. In the extreme South there is a tropical climate on the edges of the Niger River basin. The rainy season in Niger lasts three to four months, from June through September, with peak malaria transmission during the second half (August–September).

**Fig. 1:**
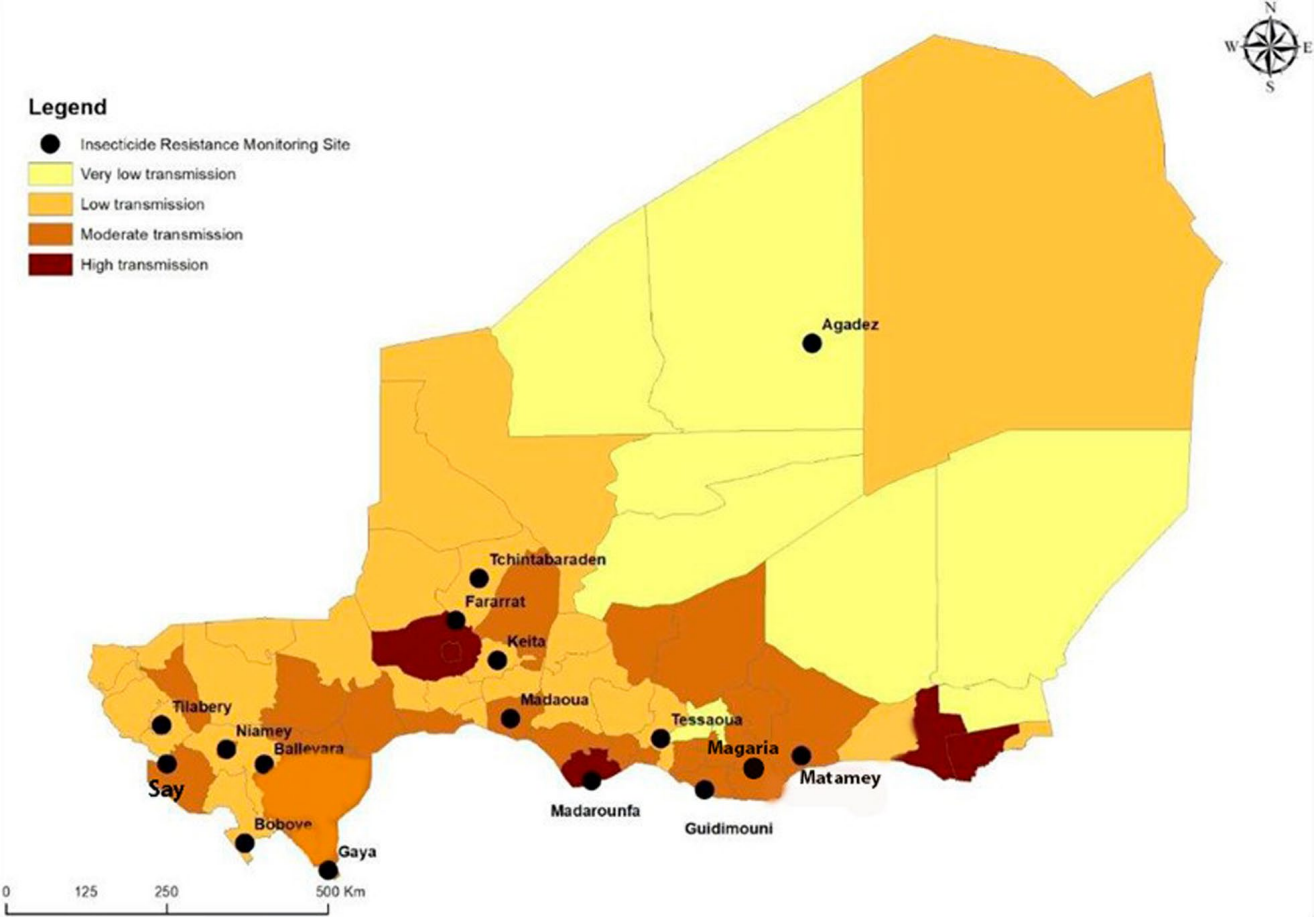
Map of Niger showing selected insecticide resistance monitoring sites across the country, 2019 to 2020

The Niger NMCP selected nine sites in 2019 and this was increased to fifteen sites in 2020 across the different endemicity zones (Fig. 1) to conduct insecticide susceptibility tests on local malaria vectors to generate data for appropriate ITN decision-making at country level. Most of the sites are in areas of intensive agriculture such as rice cultivation (particularly in Gaya, Niamey V and Tillabery), cotton growing, sugar cane, onion, and market gardening with intensive pesticide use.

### Insecticide susceptibility and resistance intensity tests

From August through December 2019 and September through December 2020, insecticide susceptibility tests were conducted once a year per site. As all vector control interventions in the country are currently based on distribution of ITNs, the priority tests were given to the pyrethroids and combinations, and chlorfenapyr insecticides.

Larvae and pupae of *Anopheles gambiae sensu lato* (*s.l*.) were collected in each site from several larval habitats, pooled, and reared to adulthood in the field laboratory. Insecticide susceptibility tests were conducted on two- to five-day-old adult females using WHO tube tests [19]. For each tube test, 80–100 female *An. gambiae s.l*. (in four tubes with 20–25 per tube) were exposed to the insecticide for one hour and mortality assessed after 24 h (Table 1). An additional 40–50 mosquitoes in two tubes were tested in parallel as controls. All tests were conducted on sites except the chlorfenapyr bottle assays that were run in the laboratory at the Centre de Recherches Médicale et Sanitaire (CERMES) under appropriate and controlled humidity and temperature condition that would not exceed 25 °C (± 2) and 70% (± 10).

**Table 1:** List of tested insecticides and concentrations

Insecticides	Diagnostic concentration (DC)	Intensity assay		Diagnostic exposure time	Delayed mortality post-exposure
		5× DC	10× DC		
Deltamethrin	0.05%	0.25%	0.50%	60 min	24 h
Permethrin	0.75%	3.75%	7.50%	60 min	24 h
Alpha-cypermethrin	0.05%	0.25%	0.50%	60 min	24 h
Piperonyl butoxide + pyrethroids	4.00%	Synergist assay		60 min synergist and 60 min pyrethroid	24 h
Chlorfenapyr	100 and 200 µg/bottles	–	–	60 min	72 h

Insecticide-impregnated papers were supplied by Universiti Sains Malaysia (USM). The diagnostic concentrations of permethrin (0.75%), deltamethrin (0.05%), alpha-cypermethrin (0.05%), were tested in all sites. When resistance to alpha-cypermethrin, deltamethrin and permethrin was confirmed (mortality below 90%), resistance intensity assays were carried out using 5 and 10 times the diagnostic concentration. Mosquitoes were exposed to the insecticides for one hour, and susceptibility was assessed according to WHO tube test procedures [19].

### Piperonyl butoxide (PBO) synergist assays

Synergist assays with PBO were conducted for deltamethrin, permethrin, and alpha-cypermethrin according to the WHO tube test protocol [19] to determine the involvement of cytochrome P450s in pyrethroid resistance. The synergist assays were conducted by pre-exposing mosquitoes to a 4% PBO paper for one hour. Mosquitoes were then transferred to tubes with one of the three pyrethroids for one additional hour of exposure. For PBO assays, controls using PBO treated papers were tested in parallel during the assays. For all tests, resistance status, synergist effect, and resistance intensity were defined following WHO criteria [19].

### Chlorfenapyr CDC bottle assays

The 250 mL Wheaton bottles were used to conduct the CDC bottle bioassays. The bottles were coated following the protocol described by Brogdon et al. [20] with 1 mL of chlorfenapyr diluted in acetone at the concentrations of 100 µg/bottle and 200 µg/bottle. These two doses were used following literature as there was no diagnostic dose for chlorfenapyr at the time of the data collections. Each dose active ingredient was pre-weighed at the U.S. Centers for Disease Control and Prevention (CDC, Atlanta, USA) to enable the coating of 50 bottles. The test was conducted following the CDC bottle assay standard testing procedures, with the exception that the tested mosquitoes were removed from the bottles after the exposure time and held in disposable cups with access to 10% sucrose solution, and mortality was scored up to 72 h [20]. Both concentrations (100 µg/bottle and 200 µg /bottle) were simultaneously tested in all nine sites in 2019 as the molecule was tested for the first time in the country and to avoid any difference of testing conditions to enable interpretation and comparison of the results of both tests with less deviation. But in 2020, only the 200 µg/bottle concentration was tested in sites where 100 µg/bottles did not achieve 98% mortality, following the previous year’s lesson learned.

### Species identification and characterization of insecticide resistance markers

A subsample of about one-hundred *An. gambiae s.l.* was randomly selected among the dead and alive population tested per site for species identification and molecular markers of resistance detection using polymerase chain reaction (PCR) analysis. DNA was extracted following the protocol described by Rudbeck et al. [21]. PCR species identification of the *An. gambiae* complex was conducted following the SINE PCR protocol described by Santolamazza et al. [22]. The presence of the L1014F knock down resistance allele was characterized using the PCR restriction fragment length polymorphism (RFLP) method as described by Martinez-Torres et al. [23] and the L1014S knock down resistance allele was characterized following the protocol described by Ranson et al. [24]. The protocol described by Weill et al. [25] was used to detect the acetylcholinesterase (*ace-*1) gene mutation.

### Statistical analysis

Insecticide resistance status was defined following WHO criteria [19], with mortality after 24 h < 90% as confirmed resistance, between 90 and < 98% as possible resistance, and ≥ 98% as susceptible. Mortality was corrected using Abbott’s formula [26] when the mortality of the control tubes was above 5% and less than 20%.

For intensity assays, corrected mortality of:

- 98–100% at 5× the diagnostic dose indicated low resistance intensity
- Less than 98% at 5× diagnostic dose implied testing 10× the diagnostic dose
- 98–100% at 10× the diagnostic dose confirmed a moderate resistance intensity
- Less than 98% at 10× the diagnostic dose indicated high resistance intensity

For the synergist assays, an increase in the mortality after pre-exposure to PBO compared to the diagnostic dose of the insecticide alone indicated the involvement of oxidase enzymes such as P450s in the resistance in the population tested. The standard deviations were calculated for both pyrethroids alone and PBO + pyrethroids using the mean mortality of each bioassay tube to estimate the statistical PBO effect difference between the insecticides alone and the synergism.

Allelic frequencies of each of the target site resistance allele was calculated in dead and alive mosquitoes and in *An. gambiae* complex species using the following formula: F (R) = (RS+2RR)/2N, where RR = total number of homozygote resistant, RS = total number of heterozygote resistant, and N = total number of mosquitoes investigated. A Fisher’s exact test was used in R 4.2.1 to test for differences between mutations genotypes in dead and alive mosquitoes and in vector species with a p-value set at 0.05% of significance.

## Results

### Pyrethroid and synergist bioassays

**Fig. 2:**
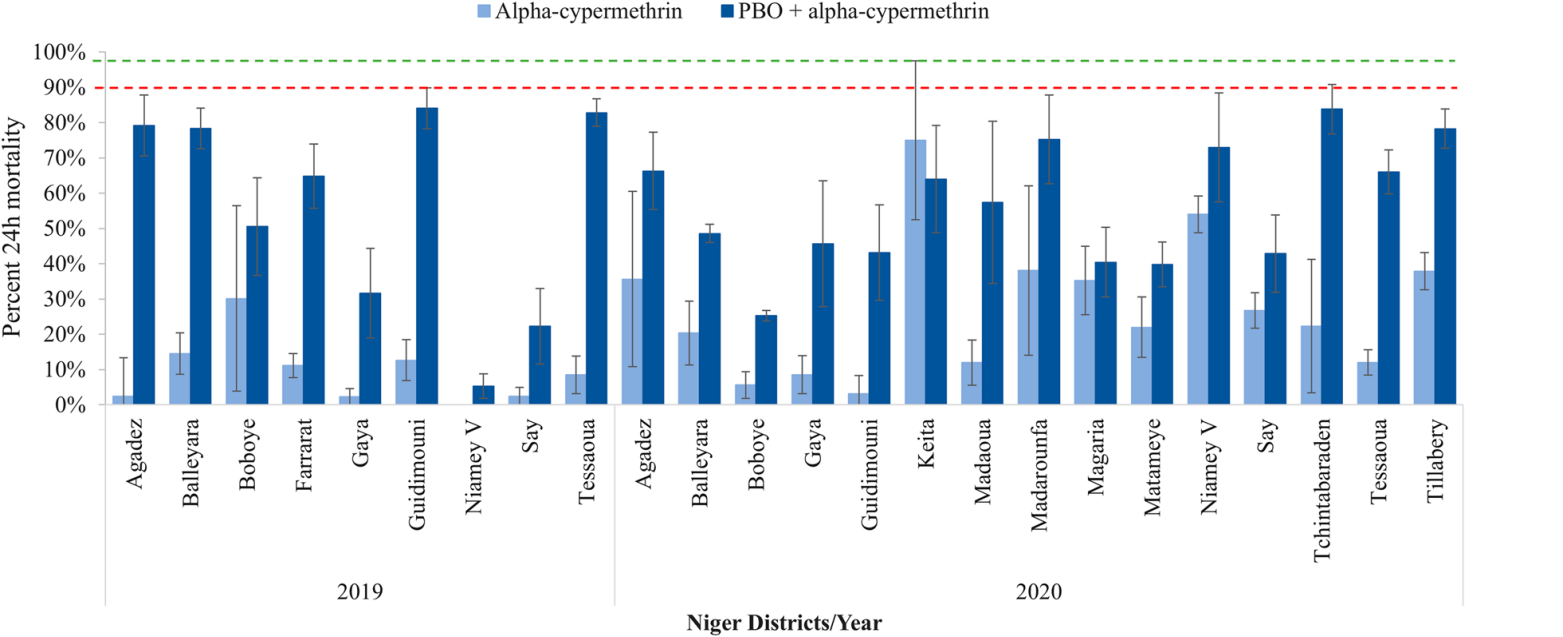
Susceptibility and synergism bioassay results of alpha-cypermethrin against *An. gambiae s.l.* in 2019 and 2020 across all sites surveyed (Error bars represent the standard deviations and, red and green dotted lines represent the WHO resistance and susceptibility threshold respectively)

**Fig. 3:**
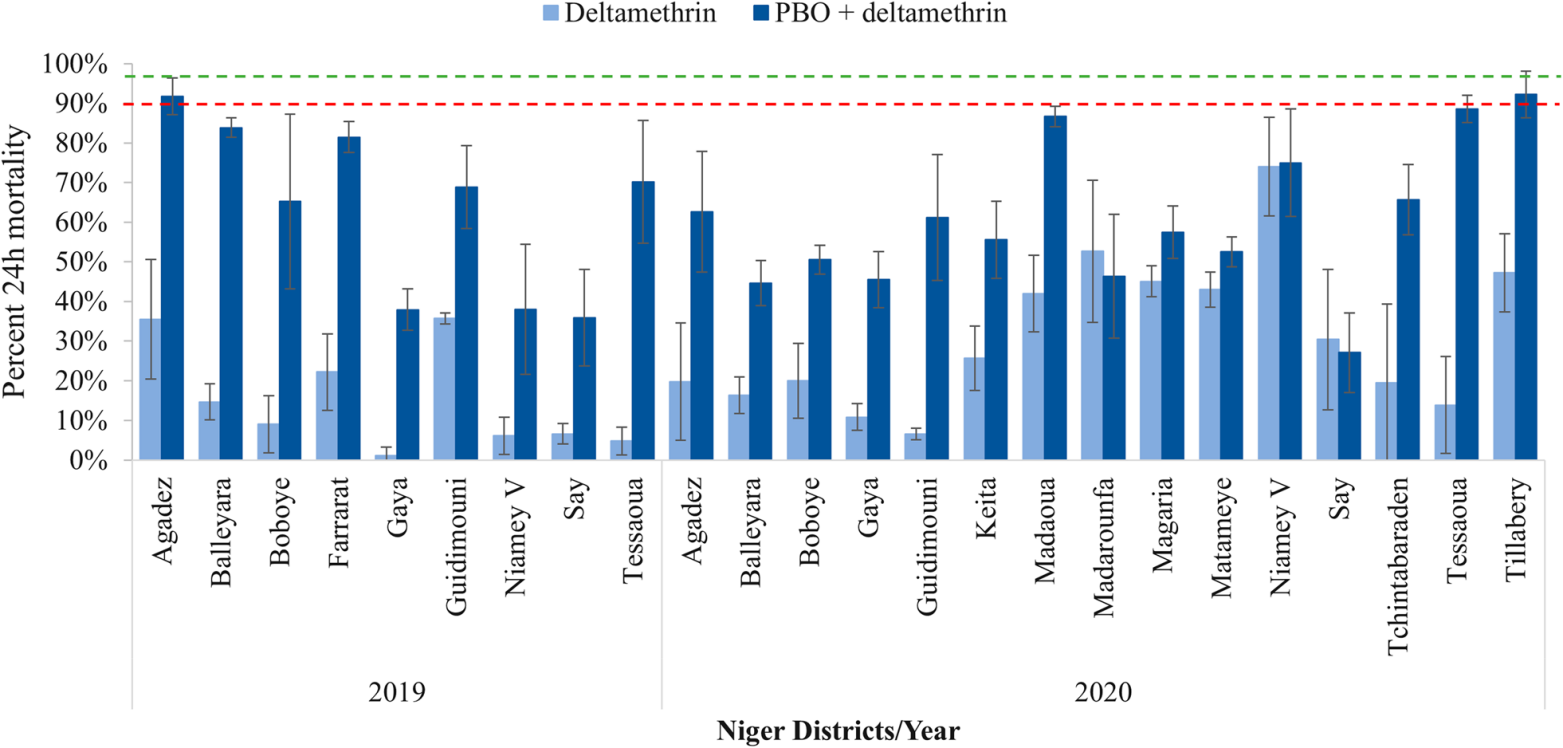
Susceptibility and synergism bioassay results of deltamethrin against *An. gambiae s.l.* recorded in 2019 and 2020 across all sites surveyed (Error bars represent the standard deviations and, red and green dotted lines represent the WHO resistance and susceptibility threshold respectively)

**Fig. 4:**
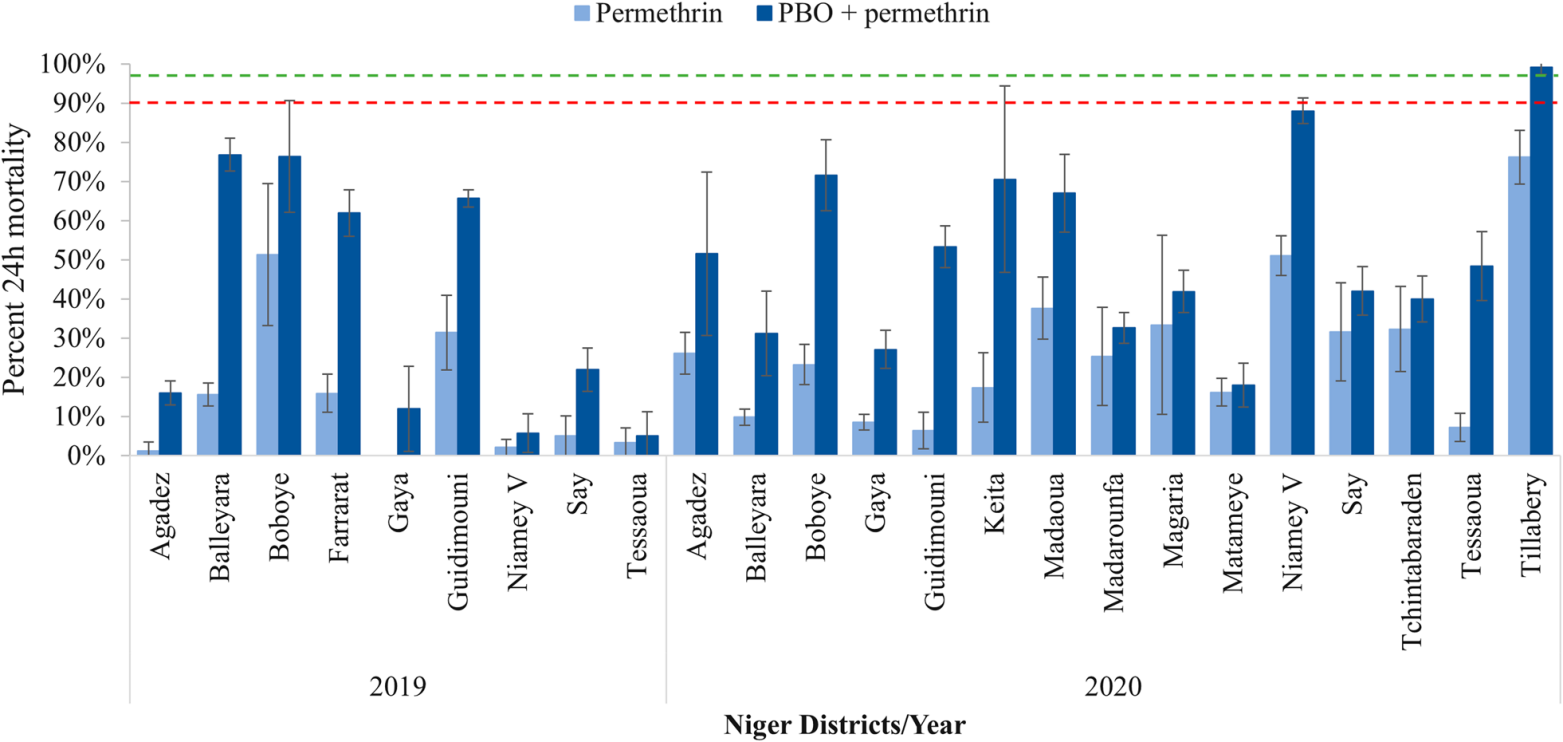
Susceptibility and synergism bioassay results of permethrin against *An. gambiae s.l.* recorded in 2019 and 2020 across all sites surveyed (Error bars represent the standard deviations and, red and green dotted lines represent the WHO resistance and susceptibility threshold respectively)

Resistance to all three pyrethroids was observed in 2019 and 2020 in all sites. Mortality against the diagnostic dose of alpha-cypermethrin (0.05%) was between 2.4%% in Agadez and 30.1% in Boboye in 2019 while the lowest mortality was observed in Guidimouni (3.2%) and the highest in Keita (75.0%) in 2020 (Fig. 2). Mortality against the diagnostic dose of deltamethrin (0.05%) was between 1.2% in Gaya and 35.7% in Guidimouni in 2019, and 6.6% in Guidimouni and 74.0% in Niamey V in 2020 (Fig. 3). For permethrin (0.75%), mosquito mortality was between 1.3% in Agadez and 51.3% in Boboye in 2019, while in 2020, the lowest mortality of (6.4%) was in Guidimouni and highest (76.2%) in Tillabery (Fig. 4).

Exposing mosquitoes to PBO before the pyrethroids did not completely restore susceptibility of the *An. gambiae s.l.* populations in any of the sites surveyed in either year, except in Tillabery where susceptibility to permethrin was recovered to 99.1% mortality. Nonetheless, in both 2019 and 2020 a substantial increase in mortality following PBO exposure was observed for pyrethroids overall and particularly for alpha-cypermethrin and deltamethrin (Figs. 2, 3, 4 and Additional file 1). Resistant mosquitoes in five sites showed no significant mortality when pre-exposed to PBO prior to exposure to pyrethroid. Magaria for alpha-cypermethrin, Madaoua and Say for deltamethrin, Madarounfa, Magaria, Say and Tchintabaraden for permethrin yielded similar mortality rates with pyrethroid alone and combined with PBO.

### Resistance intensity of *Anopheles gambiae s.l.*

In 2019, resistance intensity was tested in eight of the nine sites for alpha-cypermethrin due to limited number of mosquitoes in Fararrat, and in all nine sites for deltamethrin and permethrin. High resistance intensity was observed for all three pyrethroids except for alpha-cypermethrin and permethrin in Gaya and for alpha-cypermethrin in Tessaoua where moderate resistance was recorded.

**Fig. 5:**
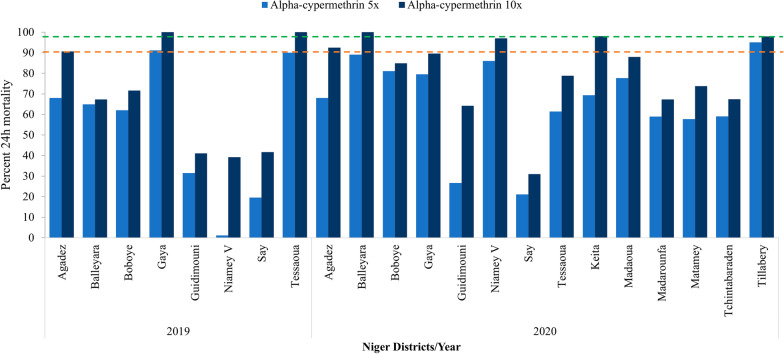
Intensity assays of alpha-cypermethrin (0.25% and 0.5%) against *An. gambiae* s.l. in 2019 and 2020 across all sites surveyed (Red and green dotted lines represent the WHO resistance and susceptibility threshold respectively)

**Fig. 6:**
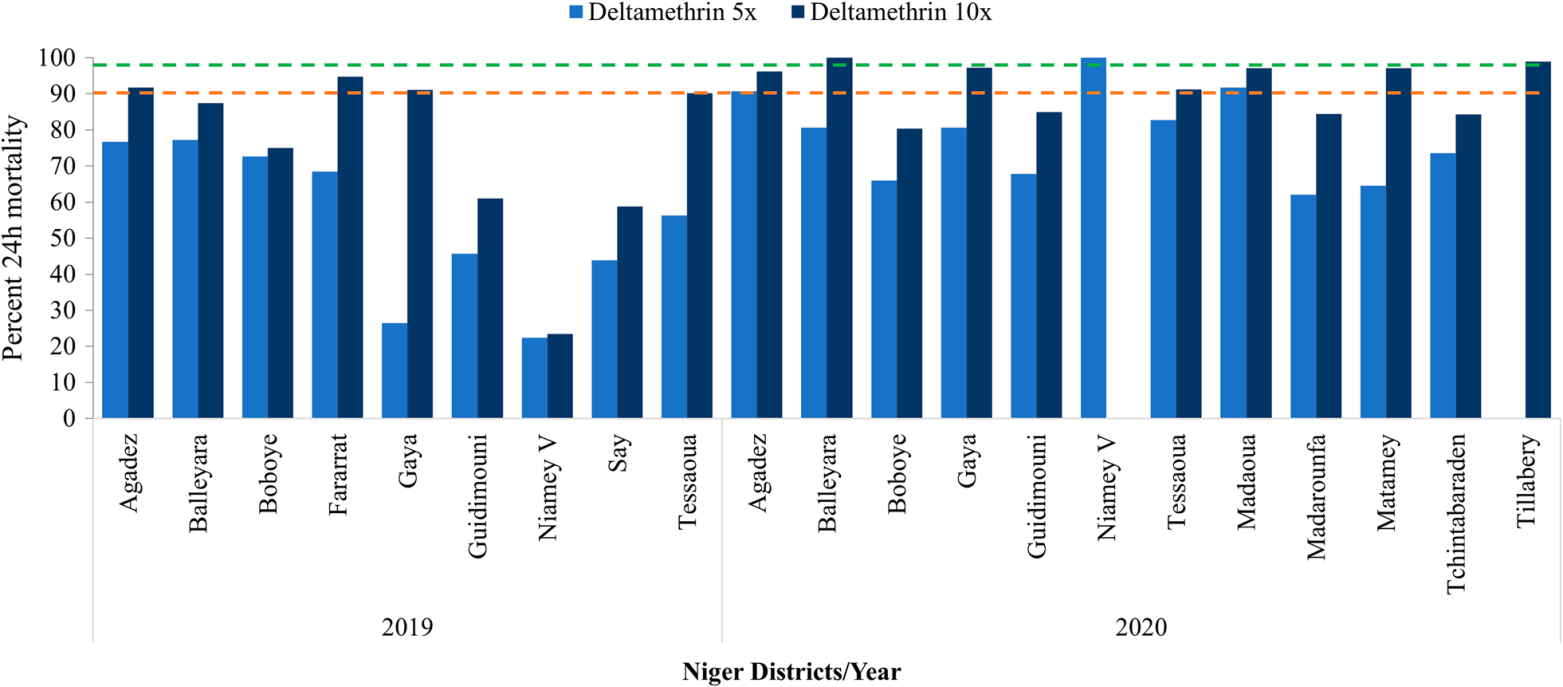
Intensity assays of deltamethrin (0.25% and 0.5%) against *An. gambiae s.l.* in 2019 and 2020 across all sites surveyed (Red and green dotted lines represent the WHO resistance and susceptibility threshold respectively)

**Fig. 7:**
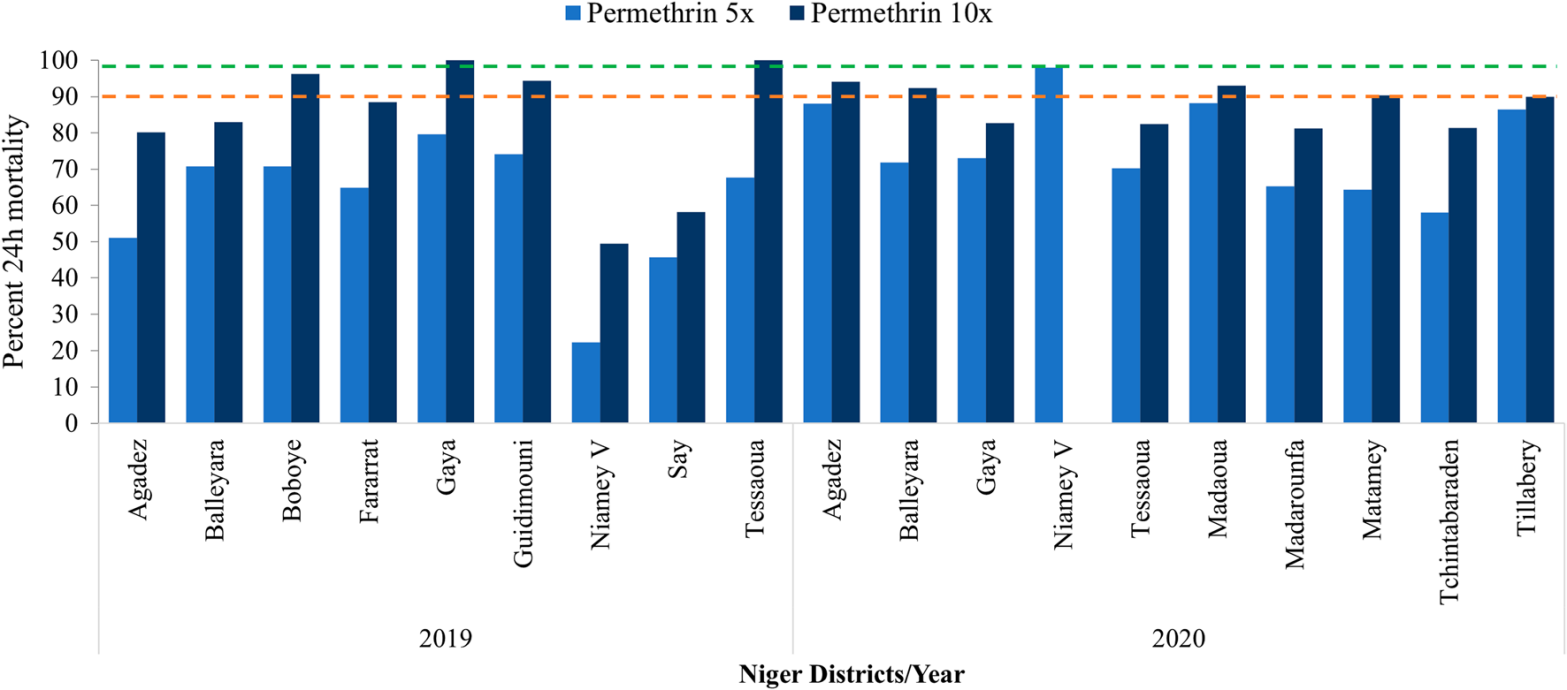
Intensity assays of permethrin (3.75% and 7.5%) against *An. gambiae s.l.* in 2019 and 2020 across all sites surveyed (Red and green dotted lines represent the WHO resistance and susceptibility threshold respectively)

In 2020, resistance intensity was tested in fourteen sites for alpha-cypermethrin, twelve for deltamethrin, and ten for permethrin due to limited availability of impregnated papers received from the supplier. The resistance intensity was also high at all sites surveyed and for all three pyrethroids except in Balleyara, Keita and Tillabery with moderate resistance for alpha-cypermethrin and deltamethrin. Only Niamey V yielded low resistance intensity against deltamethrin and permethrin with 100% mortality recorded at 5× dose for both insecticides (Figs. 5, 6, 7 and Additional file 2).

### Chlorfenapyr bioassays

**Fig. 8:**
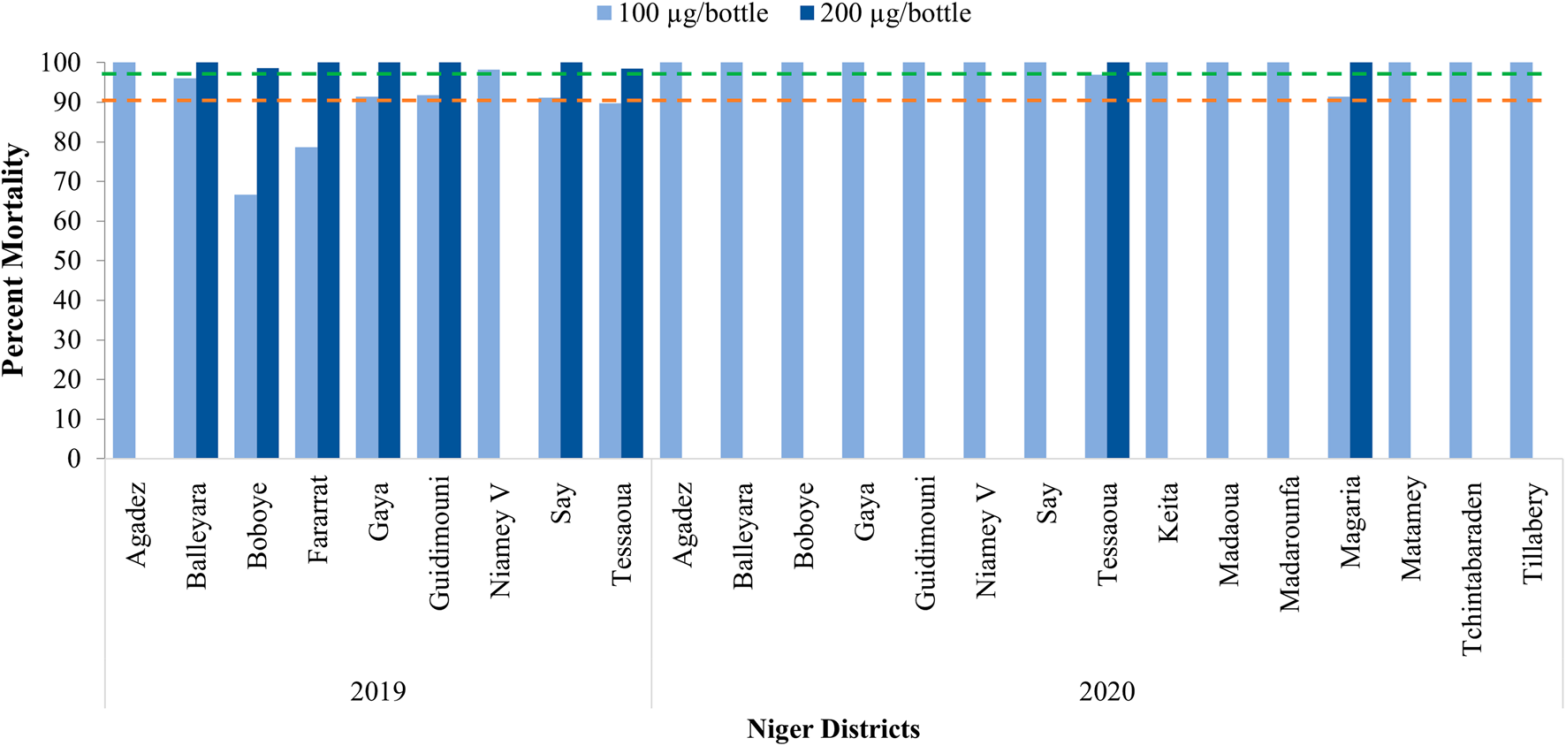
Susceptibility of *An. gambiae s.l.* to chlorfenapyr 100 µg/bottle and 200 µg/bottle in 2019 and 2020 across all sites surveyed (Red and green dotted lines represent the WHO resistance and susceptibility threshold respectively)

In 2019, mortality recorded after a 72-hour holding period showed susceptibility at the dose of 100 µg/bottle only in two sites (Agadez and Niamey V) while all sites showed susceptibility at 200 µg/bottle. In 2020, susceptibility was recorded at 100 µg/bottle in all sites except in Magaria and Tessaoua with susceptibility at 200 µg/bottle (Fig. 8 and Additional file 2).

### *Anopheles* species identification and insecticide resistance markers

A total of 1747 mosquitoes tested for susceptibility in the 15 sites were analyzed for species identification and resistance allele characterization in 2020. The resistance markers were successfully determined for 1708, 1747 and 1692 mosquitoes for *kdr-*L1014F, *kdr*-L1014S and *ace-*1 G119S, respectively while few species failed to identify.

**Fig. 9:**
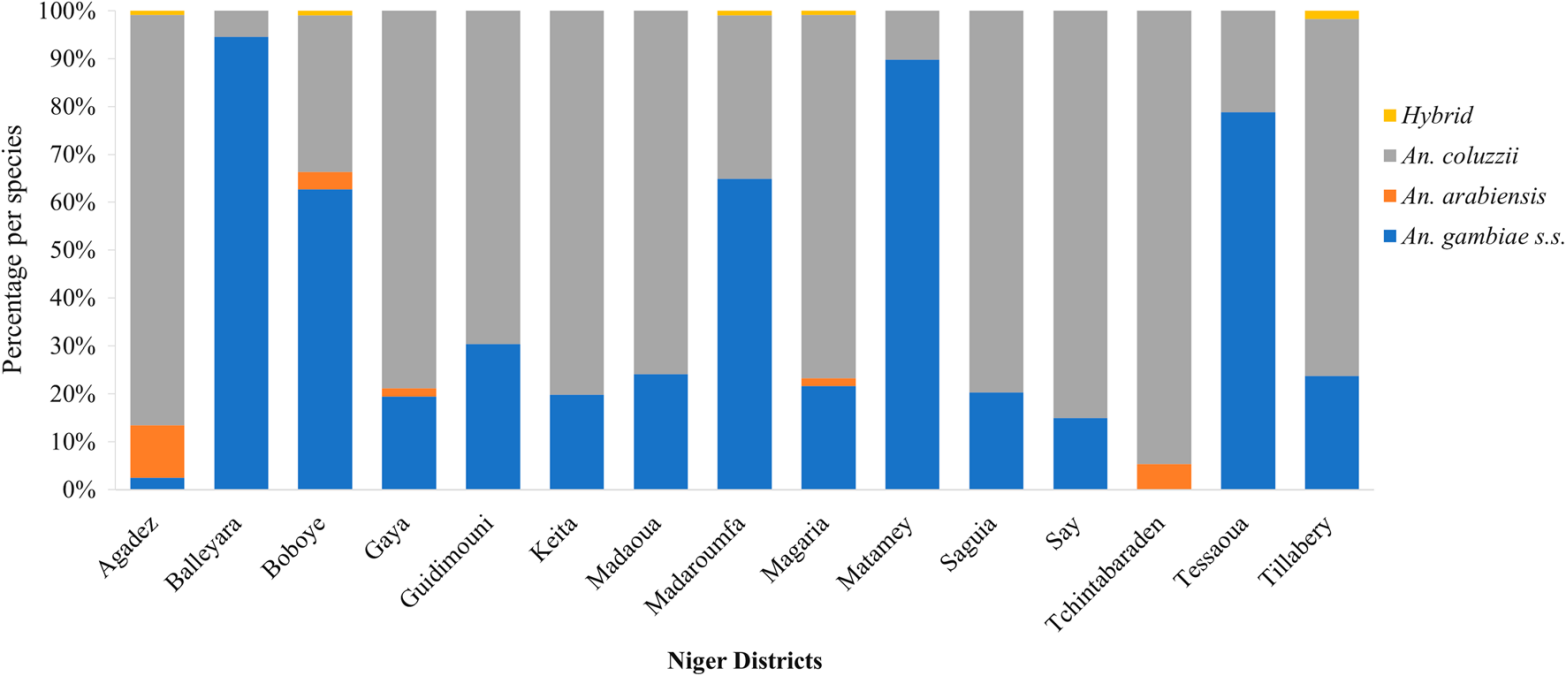
Species composition of *An. gambiae s.l.* of mosquitoes tested for insecticide susceptibility in 2020 across all sites surveyed

*Anopheles coluzzii* represented the main vector in ten of the fifteen sites with an average of 60.4% of the total mosquitoes analysed, while *An. gambiae sensu stricto* (*s.s*.) (37.7% overall) was predominantly recorded in Balleyara (94.6%), Boboye (62.7%), Madaroufa (64.9%), Matamey (89.8%) and Tessaoua (78.8%). Other members of the *An. gambiae* complex identified included *Anopheles arabiensis* (1.6%) and few hybrids of *An. coluzzii*/*An. gambiae s.s.* (0.3%) (Fig. 9).

The *kdr*-L1014F frequencies varied from 0.45 to 0.80 with a mean of 0.63 across all sites. The highest *kdr-*L1014F frequency was recorded in Gaya (0.81), followed by Tillabery (0.70), while the lowest was in Boboye (0.46). The investigation of the presence of *kdr*-L1014S showed the presence of the mutation at frequencies varying between 0.41 in Tessaoua to 0.87 in Gaya. The *ace-1* G119S mutation remained low within the populations tested with the highest frequency found in Madarounfa (0.21), followed by Niamey V (0.20) (Table 2). Furthermore, there was no significant difference of all tested target site mutations between all species of the *An. gambiae* complex, though the proportion of *An. coluzzii* was highest among the mosquitoes tested. Nevertheless, *An. arabiensis* showed lower frequency of all the three mutations compared to *An. coluzzii* and *An. gambiae s.s.* (Table 2). On the other hand, there was not significant difference between the overall allelic frequency of the *kdr* L1014F (*p* = 0.122) and *ace*-1 G119S (*p* = 0.187) mutations among the dead and the live mosquitoes tested while the L1014S mutation allele frequency was higher among the dead mosquitoes than the survivals from the susceptibility testing (*p*= 0.025). Specifically, higher L1014F frequency was observed within the live mosquitoes compared to the dead in Guidimouni (*p* = 0.002) while the reverse trend was observed in Tillabery (0.031). Similarly, higher L1014S frequency among the dead mosquito population in Keita (*p* = 0.002). The *ace-1* G119S showed also variable frequencies among the tested mosquitoes with higher G119S mutation frequency among the live mosquitoes in Balleyara (*p* = 0.008) and reverse trend observed in Tessaoua (*p* = 0.005) (Table 3 and Additional file 3).

**Table 2:** Number of mosquitoes tested and frequency of target site resistance mutations per species

Species	*Kdr*-L1014F					*Kdr*-L1014S					*Ace*-1 G119S				
	Total	RR	RS	SS	Freq	Total	RR	RS	SS	Freq	Total	RR	RS	SS	Freq
*An. arabiensis*	26	11	5	10	0.52^a^	27	11	3	13	0.46^bc^	27	5	0	22	0.09^a^
*An. coluzzii*	1017	506	255	256	0.62^a^	1036	687	112	237	0.72^ab^	999	132	18	849	0.14^a^
*A. gambiae s.s*	630	314	155	161	0.62^a^	647	373	108	166	0.66^ab^	632	80	8	544	0.13^a^
Hybrid *An. coluzzii/An. gambiae* s.s	6	3	2	1	0.67^a^	6	5	0	1	0.83^bc^	5	1	0	4	0.10^a^
Total	1679	834	417	428	0.62	1716	1076	223	417	0.69	1663	218	26	1419	0.14

Frequency of the target resistance alleles was described for different species of the *An. gambiae* complex and compared using statistical analysis

*Freq* frequency, *RR* homozygous resistant, *RS* heterozygous resistant, *SS* homozygous susceptible

^a^All frequencies are not significantly different

^b^All frequencies are significantly different

^c^Both frequencies are significantly different

**Table 3:** Number of mosquitoes tested and frequency of target site insecticide resistance alleles across the sites in 2020

Sites		Resistance alleles																				
		*Kdr-L1014F*							*Kdr-L1014S*							*Ace-1 G119S*						
		Total	RR	RS	SS	Freq/status	p value	Freq	Total	RR	RS	SS	Freq/status	p value	Freq	Total	RR	RS	SS	Freq/status	p value	Freq
Agadez	Dead	44	27	5	12	*0.67*	0.211^*^	*0.62*	43	28	1	14	*0.66*	0.778^*^	*0.65*	42	6	1	35	*0.15*	0.591^*^	*0.17*
	Alive	70	38	6	26	*0.59*			75	43	10	22	*0.64*			72	13	1	58	*0.19*		
Balleyara	Dead	45	24	12	9	*0.67*	0.773^*^	*0.65*	45	27	6	12	*0.67*	0.331^*^	*0.63*	45	8	1	36	*0.19*	***0.008***^***#***^	*0.12*
	Alive	63	34	13	16	*0.65*			70	32	20	18	*0.6*			63	4	0	59	*0.06*		
Boboye	Dead	43	10	25	8	*0.52*	0.169^*^	*0.46*	44	32	3	9	*0.76*	0.121^*^	*0.81*	43	8	0	35	*0.19*	0.583^*^	*0.17*
	Alive	70	6	47	17	*0.42*			71	55	10	6	*0.85*			72	11	0	61	*0.15*		
Gaya	Dead	43	32	5	6	*0.80*	0.594^*^	*0.81*	45	38	4	3	*0.89*	0.558^*^	*0.87*	40	3	0	37	*0.08*	1.000^*^	*0.08*
	Alive	73	56	10	7	*0.84*			75	61	7	7	*0.86*			72	5	1	66	*0.08*		
Guidimouni	Dead	53	22	13	18	*0.54*	***0.002***^***#***^	*0.64*	53	37	10	6	*0.79*	0.750^*^	*0.78*	51	6	0	45	*0.12*	0.084^*^	*0.13*
	Alive	62	41	9	11	*0.73*			62	44	7	11	*0.77*			60	8	0	52	*0.13*		
Keita	Dead	54	18	24	12	*0.56*	0.107^*^	*0.61*	55	48	2	5	*0.89*	***0.002***^***#***^	*0.80*	55	8	1	46	*0.15*	0.443^*^	*0.13*
	Alive	62	32	18	12	*0.66*			65	45	5	15	*0.73*			60	7	0	53	*0.12*		
Madaoua	Dead	49	20	13	16	*0.54*	1.000^*^	*0.55*	50	39	7	4	*0.85*	0.851^*^	*0.86*	49	2	0	47	*0.04*	***0.023***^***#***^	*0.08*
	Alive	70	27	22	21	*0.54*			70	58	5	7	*0.86*			70	9	0	61	*0.13*		
Madarounfa	Dead	46	21	4	21	*0.50*	0.103^*^	*0.57*	46	22	7	17	*0.55*	0.411^*^	*0.59*	46	8	0	38	*0.17*	0.253^*^	*0.21*
	Alive	67	36	10	21	*0.61*			67	38	6	23	*0.61*			67	16	0	51	*0.24*		
Magaria	Dead	68	41	13	14	*0.70*	0.333^*^	*0.67*	68	42	9	17	*0.68*	0.581^*^	*0.67*	68	5	2	61	*0.09*	0.824^*^	*0.09*
	Alive	52	25	16	11	*0.63*			52	29	9	14	*0.64*			51	5	0	46	*0.10*		
Matamey	Dead	46	26	12	8	*0.70*	0.039^*^	*0.61*	46	30	7	9	*0.73*	0.544^*^	*0.75*	45	7	0	38	*0.16*	1.000^*^	*0.16*
	Alive	69	33	11	25	*0.56*			74	52	9	13	*0.76*			69	11	0	58	*0.16*		
Niamey V	Dead	31	16	7	8	*0.63*	1.000^*^	*0.63*	30	16	7	7	*0.65*	0.877^*^	*0.64*	31	3	3	25	*0.15*	0.265^*^	*0.20*
	Alive	85	42	24	19	*0.64*			84	41	25	18	*0.64*			83	15	6	62	*0.22*		
Say	Dead	38	22	9	7	*0.70*	0.104^*^	*0.61*	39	27	8	4	*0.79*	0.112^*^	*0.73*	39	4	1	34	*0.12*	0.190^*^	*0.08*
	Alive	67	29	19	19	*0.57*			68	44	6	18	*0.69*			67	3	2	62	*0.06*		
Tchintabaraden	Dead	37	18	10	9	*0.62*	0.085^*^	*0.54*	40	20	3	17	*0.54*	0.407^*^	*0.50*	36	5	0	31	*0.14*	0.562^*^	*0.16*
	Alive	71	23	24	24	*0.49*			75	34	3	38	*0.47*			73	13	0	60	*0.18*		
Tessaoua	Dead	43	25	7	11	*0.66*	0.481^*^	*0.63*	42	11	11	20	*0.39*	0.782^*^	*0.41*	44	2	2	40	*0.07*	***0.005***^***#***^	*0.15*
	Alive	71	40	7	24	*0.61*			75	25	13	37	*0.42*			71	13	4	54	*0.21*		
Tillabery	Dead	49	34	8	7	*0.78*	0.031^*^	*0.70*	50	36	1	13	*0.73*	0.095^*^	*0.67*	47	7	0	40	*0.15*	0.417^*^	*0.13*
	Alive	67	36	14	17	0.64			68	42	1	25	*0.63*			64	6	2	56	*0.11*		
Total	Dead	689	356	167	166	*0.64*	0.122^*^	*0.62*	696	453	86	157	*0.71*	*0.025*^*#*^	*0.69*	681	82	11	588	*0.13*	0.187^*^	*0.14*
	Alive	1019	498	250	270	0.61			1051	643	136	272	0.67			1014	139	16	859	0.15		

Mosquitoes analyzed were randomly selected among the dead and live mosquitoes after insecticide susceptibility tests. The frequency between dead mosquitoes and live mosquitoes were compared using statistical analysis test

^*^All frequencies are not significantly different

^#^All frequencies are significantly different between dead and live mosquitoes

## Discussion

Resistance of *An. gambiae s.l.* to the diagnostic dose of the three pyrethroids tested (deltamethrin, permethrin, and alpha-cypermethrin) was observed in 2019 and 2020 in all sites in Niger. Both consecutive monitoring years recorded similar resistance status on *Anopheles gambiae s.l.* at each site surveyed twice. The presence and increase in insecticide resistance alleles is a known trend in most African countries [27–35]. Most sub-Saharan African countries, including Niger, have reported resistance, particularly to pyrethroid insecticides [16, 17]. As further reported by several authors, the spread and increasing insecticide resistance of malaria vectors is partly due to the use of insecticide-based tools for public health and partly due to agricultural applications of pesticides [31, 36–38]. Niger has conducted four mass ITN distribution campaigns using pyrethroid-only ITNs in high malaria burden districts to gradually cover the entire country with ITNs. Mass ITN distributions targeting those districts started in 2005, while mass distribution and universal coverage of selected regions started in 2014, using pyrethroid-only treated ITNs. Furthermore, most of the sites selected for insecticide resistance monitoring in Niger are in areas of intensive agriculture including rice and cotton cultivation, with intensive pesticide use, which may have contributed to the moderate or high pyrethroid resistance intensity recorded in most of the sites. This presents an obvious challenge for malaria control as it limits the country’s options for efficient insecticide-based vector control interventions [10, 11].

Synergist assays showed the impact of PBO in substantial increased of mortality for all pyrethroids in nearly all sites, indicating the involvement of P450 enzymes as an important resistance mechanism. Per WHO recommendations, the deployment of PBO ITNs is recommended when a vector population is highly resistant to pyrethroids, and a significant increase of mortality is observed when those vectors are pre exposed to PBO [39]. As several PBO ITNs are now available, the decision to use them should be evidence-based and driven by the percentage increment of mortality in the presence of PBO. Therefore, PBO ITNs could be procured and distributed strategically in Niger. The data gathered across the country showed substantially higher mosquito mortality against alpha-cypermethrin and deltamethrin than permethrin after pre-exposure to PBO in most of the sites. This trend could support for the choice of alphacypermethrin or deltamethrin-based PBO ITNs to be prioritized in most of the sites. The use of PBO ITNs with deltamethrin or alpha-cypermethrin-based combination have shown higher performance than those with permethrin, as reported by several authors over studies particularly conducted in Western African countries where the vector populations are highly resistant to pyrethroids [28, 40]. However, all PBO-ITNs have proven to be more effective that the pyrethroid-only ITNs [41]. Given the observed effect of PBO at all sites, the NMCP could prioritize PBO-based ITNs in the country’s malaria vector control management plan.

Susceptibility to chlorfenapyr in all sites in 2019 and in 2020 with the doses of 100 and 200 µg/bottles confirms the suitability of chlorfenapyr as an option for controlling highly pyrethroid-resistant vector populations as previously recorded [42–44]. The overall data on chlorfenapyr susceptibility was similar to several reports from studies conducted in sub-Saharan Africa [28, 45]. Kouassi et al. [28] reported in 2020 that higher mortality of mosquitoes could be observed using chlorfenapyr, particularly in areas where insecticide detoxification was the main resistance mechanism, suggesting that ITNs with chlorfenapyr may be appropriate in Niger.

Multiple resistance mechanisms are associated with insecticide resistance in mosquitoes, including target-site mutations and markers of metabolic and cuticular resistance [46, 47]. Among these, resistance to pyrethroids and dichlorodiphenyltrichloroethane (DDT) is associated with a substitution of the amino acid leucine with either phenylalanine (L1014F) or serine (L1014S) at position 1014 on the voltage-gated sodium channel gene [30, 35, 46–50]. For organophosphate and carbamate insecticides, a target site mechanism, known as *ace*-1, represents the substitution of an amino acid glycine to serine at position 119 on the acetylcholinesterase 1 gene (G119S) [25]. All of these mutations can occur within a single mosquito, contributing to resistance to multiple classes of insecticides [51].

Like most of the West African countries, three species of the *An. gambiae* complex were determined across the country with *An. coluzzii* as predominant species among the sites surveyed and *An. arabiensis* in few sites [52]. Furthermore, similar and higher frequency of all target site mutations was observed for *An. coluzzii* and *An. gambiae s.s.* compared to *An. arabiensis*. This study also showed that, the frequency of the *kdr*-L1014F mutation observed among dead and live mosquitoes after insecticide susceptibility testing was overall similar across all sites in Niger, while the L1014S was slightly higher within the dead mosquitoes. This showed that both mutations were well established in the country within the predominant vectors and calls for appropriate vector control strategies and insecticide management plan. The data corroborates with other country reports [53–55] and confirms the finding of Donnelly et al., showing that the phenotypic status of a resistant population may not be directly correlated with the genotype [56], whereby it could be expected higher allele frequency of the mutation among surviving mosquitoes from an insecticide susceptibility test than the dead. Since the work of Czeher et al. [16] reporting earlier in 2005 the presence of the L1014F mutation within the population of *An. coluzzii* after the first pyrethroid-ITN distribution in the country, limited entomological data were available in Niger before recent works conducted by Soumaila et al. [17] and Ibrahim et al. [18] confirming the presence of the L1014F mutation in the country following resistance observed in selected sites while the L1014S mutation had not previously been investigated.

This study characterized the presence of the L1014S allele for the first time in Niger, and surprisingly, the frequency of the mutation was already high within the local populations, showing that the mutation may have been occurring in the country for an unknown period, in contrast to other countries where only a few specimens carrying this allele have been recorded [57, 58]. Similar to the L1014 mutations, the *ace-*1 G119S resistance allele was also detected within the mosquito populations in all sites, but at a low frequency. Even though carbamate and organophosphate insecticides are not reported in this study, the frequency of the *ace-*1 G119S mutation suggests that the mutation was also present and any pressure on the vectors using carbamate or organophosphate-based insecticides will contribute to the increase of the mutation frequency, therefore reducing the range of insecticides that may be available for malaria vector control interventions in the country.

## Conclusion

Findings from this study showed malaria vector resistance to pyrethroids and susceptibility to chlorfenapyr, in addition to a positive effect of PBO pre-exposure on vector mortality in all sites. As prevention measures to control malaria in Niger focus on the universal coverage of ITNs, the threat of insecticide resistance and multiple resistance mechanisms could be overcome by strategic management of the issue using available tools. The data collected over the two years showed that PBO-based ITNs and ITNs that include chlorfenapyr could be advocated for effective malaria vector control.

## Supplementary Information

**Additional file 1. **Piperonyl butoxide synergism data.

**Additional file 2. **Chlorfenapyr and pyrethroid intensity data.

**Additional file 3. **PCR species identification and insecticide resistance mutation allele data.

## Data Availability

All data generated or analyzed during this study are included in this published article and its supplementary information files.

## Abbreviations

*ace-*1: Acetylcholinesterase
CDC: Centers for Disease Control and Prevention
IRS: Indoor residual spraying
ITN: Insecticide-treated net
*kdr*: Knocked-down resistance
NMCP: National Malaria Control Programme
PBO: Piperonyl Butoxide
PCR: Polymerase chain reaction
SINE: Short interspersed element
WHO: World Health Organization
